# Person-centered care during childbirth and associated factors among mothers who gave birth at health facilities in Hawassa city administration Sidama Region, Southern Ethiopia

**DOI:** 10.1186/s12884-022-04909-3

**Published:** 2022-07-22

**Authors:** Sewunnet Azezew Getahun, Abebaw Abeje Muluneh, Wudit Wassu Seneshaw, Sewnet Getaye Workie, Zemenu Yohannes Kassa

**Affiliations:** 1 Department of Midwifery, School of Nursing and Midwifery, College of Medicine and Health Sciences, Debre Berhan University, Debre Berhan, Ethiopia; 2 Department of Midwifery, College of Medicine and Health Sciences, Hawassa University, Hawassa, Ethiopia; 3 Department of Midwifery, College of Medicine and Health Sciences, Wachamo University, Hosanna, Ethiopia; 4 Department of Public Health, College of Medicine and Health Science, Debre Berhan University, Debre Berhan, Ethiopia

**Keywords:** Person-centered care during childbirth, Hawassa, Ethiopia

## Abstract

**Introduction:**

Person-centered care is a pivotal component of strategies to improve the utilization of maternity care during childbirth. However, there is limited information on the level of person-centered care during childbirth in Ethiopia. Therefore, this study aimed to assess the level of person-centered care during childbirth and associated factors in Hawassa city administration health facilities Sidama region, Ethiopia.

**Method:**

Institutional based cross-sectional study was conducted among randomly selected health facilities in Hawassa city administration from September 5 to October 30, 2021. A multistage sampling technique was employed to enroll the study participants. Data were collected through face-to-face interviews using a structured questionnaire. After data collection, it was checked for completeness and consistencies then coded and entered into Epi data version 4.4.2 and exported to SPSS version 25 for analysis. Descriptive statistics were generated to describe the study findings. Then simple and multivariable linear regressions were computed. All predictor variables with *P*-value ≤ 0.25 in the simple linear regression were fitted into the multivariable linear regression model and a *P* < 0.05 was considered statistically significant. Finally, the result of the study was presented in texts, tables, and figures.

**Result:**

The mean score of person-centered care during childbirth in Hawassa city was 56 with 95% of CI: [55.1, 57] and with SD ± 11.2. Giving birth at private health institutions (β = 4.3, 95% CI: (2.37, 6.22) and childbirth which was attended by a care provider who had provided ANC for mothers initially (β = 5.48, 95% CI: 3.15, 7.81) had significantly higher positive betas on person-centered care during childbirth. However, mothers who didn’t get a formal education (β = -3.00, 95% CI: (-5.27, -.73) and mothers with a dead pregnancy outcome (β = -7.04, 95% CI: -10.4, -3.66) decreases the person-centered care during childbirth.

**Conclusion:**

This paper showed that person-centered care during childbirth was low compared with other studies. It shall be beneficial if the city administration health facilities implement midwife-led care to improve person-centered care during childbirth.

## Background

Person-centered care during childbirth is defined as providing maternity care is respectful and responsive ways based on women’s preferences, needs, and values before, during, and after childbirth [1, 2]. It is a pivotal component of strategies to improve the utilization of maternity care during childbirth [3, 4]. It is a key domain of the quality of maternal health services [5, 6]. Person-centered care (PCC) during childbirth comprises three domains of patient experience: namely autonomy and communication, respect and dignity, and supportive care [5, 6].

Furthermore, its main advantage is to avoid mistreatment, abuse, disrespect, and neglect of women during childbirth in health facilities [7, 8]. World Health Organization adopted integrated person-centered Health services and developed five key strategies to make health services more person-centered [9]. Moreover, priority was given its application to maternal and newborn health services [3, 4].

The lack of person-centered care is a common problem in maternal health care [10, 11]. Studies showed that women are not getting adequate person-centered care during childbirth in low and middle-income countries [6, 12]. Lack of person-centered care during childbirth leads to poor community perceptions of quality of care which can infringe trust between women and healthcare providers and deters women from seeking maternal health care services in low resource setting countries [13, 14]. Status of the health care system, shortages in human resources, and commodities were mentioned as contributors to the lack of person-centered care during childbirth [10].

Adequate evidence, though it is limited, is very important for policy makers and public health experts to improve person-centered care during childbirth which can intern improve the quality of maternity care and maternal mortality. So, this study was designed to assess the level of person-centered care during childbirth and associated factors in Hawassa city administration by using the newly validated PCC during childbirth scale measurement items for middle- and low-income countries.

##  Methods and materials

### Study settings and design

An institutional-based cross-sectional study was conducted in Hawassa city administration from September 5 to October 30, 2021, among mothers who gave birth in Hawassa city health facilities. Hawassa is the administrative City of the Sidama region and located 275 km south of Addis Ababa. According to the 2021 City Health Department estimation report, 394,057 people were living in Hawassa of which 91,815 werewomen in the reproductive age group. Hawassa city has eight sub-cities and 32 kebeles. A total of 86health care facilities (one public comprehensive and specialized hospital, one general public hospital, one public primary hospital, four private hospitals, 11 public health centers, 17 health posts and 52 private clinics) have been providing service in the city. Hawassa University’s comprehensive and specialized hospital is the largest hospital in southern Ethiopia which renders service in the region and the neighboring region. Three public health hospitals and four private health facilities are providing comprehensive essential obstetric care in the city. The remaining 11 public health centers are giving basic essential obstetric care.

### Population

All randomly selected women who gave birth at selected health facilities in Hawassa city during the study period were included in this study. However, mothers who were referred to other health institutions before completing the service due to emergency conditions were excluded from the initial health facility.

### Sample size and sampling procedure

The sample size for this study was determined using a single population proportion formula with the assumption of the standard normal distribution corresponding to a 95% confidence interval, 5% margin of error, 64.5% proportion of person-centered care during childbirth in Dessie city, Northeastern Ethiopia [15]. By considering 1.5 design effects and10% possible non-response rate the final sample size was 581.

A multistage sampling technique was employed to enroll the study participants. Initially, Hawassa city administration’s health facilities were stratified into public and private health facilities. Then, 5 public and 3 private health facilities were randomly selected and the sample size was distributed proportionally for each selected health facility depending on the total estimated case flow. Finally, systematic random sampling technique was used to draw study participants from each selected health facility.

### Operational definitions

#### Person-centered care during childbirth

Person-centered care during childbirth is measured using the PCC during childbirth scale, which has three domains: dignity and respect, communication and autonomy, supportive care, and 30 items with a four-point response scale. i.e., 0 “(“no, never””), 1 “(“yes, a few times””), 2 “(“yes, most of the time””),and 3 “(“yes all the time””) [5, 6, 16], and with negative items reverse coded so that high numbers represent good care). Therefore, the scale score ranges from zero to 90.

#### Dignity and respect

Measured by six items with each four-point response scale, i.e., 0 “(“no, never””), 1 “(“yes, a few times””), 2 “(“yes, most of the time””), and 3 “(“yes, all the time””) so, the total score ranges from 0 to 18.

#### Communication and Autonomy

Measured by using nine items with each item has a four-point response scale, i.e., 0 “(“no, never””), 1 “(“yes, a few times””), 2 “(“yes, most of the time””), and 3 “(“yes, all the time””). So, the total score ranges from 0 to 27.

#### Supportive care

Measured by using 15 items with each item has a four-point response scale, i.e. 0 “(“no, never””), 1 “(“yes, a few times””), 2 “(“yes, most of the time””) and 3 “(“yes, all the time””). So, the total score ranges from 0 to 45**.**

Full PCMC and each sub-scale are categorized into “low, medium, and high”. Low was defined as scores in the approximate lower 25th percentile and scores in the top 75th percentile were defined as high by Dr. Patience A Afulani and colleague [16].

#### Data collection tools and procedure

A pretested, structured, interviewer tool was used. The tool used to collect socio-demographic, obstetric history, health facility, and health care provider related data was prepared by reviewing different articles. A newly validated standard tool in Kenya and India to measure the person-centered maternity care during childbirth scale for middle and low-income countries was used for the person-centered care during childbirth part [5, 6]. This scale includes 30 items that span three domains: dignity and respect (6 items), communication and autonomy (9 items), and supportive care [15] items. First, the questionnaire were prepared in English then translated into local language, Amharic. The scale has good internal consistency reliability, with Cronbach’s alpha above 0.80; and high content, construct, and criterion validity. Internal reliability for this study was checked by calculating Cronbach’s alpha for the full scale and each of the domains and it was found 0.78 for dignity and respect, 0.82 for communication and autonomy, 0.84 for supportive care sub-scales, and 0.88 for full scale.

Four BSc midwives and two MSc midwives were enrolled as data collectors and supervisors respectively. Data collection was made through face-to-face interview when clients were discharge to home.

#### Data quality assurance

A pretest was done on 29 (5% of the sample size) at Yirgalem General Hospital before the actual data collection period to check whether the questionnaires were simple, clear, and easily understandable and necessary modifications were made accordingly. One day training was given for both data collectors and supervisors concerning the data collection processes and data handling. During the data collection period, data were checked for completeness and consistency.

#### Data processing and analysis

The data was coded, cleaned, and entered to Epi data version 4.4.2 and exported to SPSS version 25 for analysis. Descriptive statistics were generated to describe the findings. Then simple and multivariable linear regression analysis was fitted to identify the factors associated with person-centered care during childbirth. Before fitting the linear regression model, the assumption of linearity was checked using a scatter plot. The normality of continuous data was checked by plotting histograms and Q-Q plots. And the assumption of multicollinearity was checked by the Variance Inflation Factor (VIF) (acceptable range is less than 10) and tolerance test (acceptable range is greater than 0.1). So, for this data, the maximum VIF value was 2.4 and the minimum value of the tolerance test was 0.4. The Durbin Watson statistic (acceptable range is 1.5 to 2.5) was used to check the assumptions that the residual values are independent. Hence, the value of the Durbin Watson statistic for this data was 1.79.

The model fitness was checked by multiple correlation coefficients and ANOVA test significance. The value of multiple correlation coefficients for this data was 0.64 and the *p*-value of the ANOVA test was < 0.001. Then Simple and multiple linear regression analysis were fitted after creating dummy variables. And those factors found with their *P*-value ≤ 0.25 in the simple linear regression were fitted into the multivariable regression model, and *P* < 0.05 was considered statistically significant. Finally, the result of the study was presented in tables, figures, and texts based on the data obtained.

## Result

### Socio-demographic characteristics of respondents

Five hundred sixty-four mothers have participated in this study with a response rate of 97.1%. Of the total number of women who agreed to participate in the study, 265 (47%) participants were 25–29 years. The mean age of respondents was 27.61 (SD ± 4.635) years. Nearly three-quarters of study participants, 395 (70%), lived as urban dwellers. Regarding respondents’ partners level of education, 291 (52.3%) of mothers’ partners attended college and above. The respondents’ estimated median monthly household income was 6200 Ethiopian Birr (Table 1).

**Table 1:** Socio-demographic characteristics of mothers who gave birth in Hawassa city health facilities, Southern Ethiopia, 2021 (*n* = 564)

Variable	Category	Frequency	Percentage (%)
Age	18–24	121	21.5
	25–29	265	47.0
	30–34	110	19.5
	35–39	68	12.1
Residence	Urban	395	70.0
	Rural	169	30.0
Marital status	Currently in marital union	548	97.2
	Not in marital union ^a^	16	2.8
Women education	Non-formal education	114	20.2
	Primary school	148	26.2
	Secondary school	125	22.2
	College and above	177	31.4
Women occupation	Housewife	289	51.2
	Government-employed	101	17.9
	Self-employed	70	12.4
	Merchant	67	11.9
	Student	31	5.5
	Others^b^	6	1.1
Partner occupation	Government employee	218	38.7
	privet employee	114	20.2
	Merchant	87	15.4
	Farmer	95	16.8
	Daily laborer	34	6.0
	Other^c^	8	1.4

NB: ^a^Cohabitant, single, widowed, and divorced, ^b^ unemployed &daily laborer, ^c^ Student& unemployed

### Obstetric and health facilities characteristics of respondents

The result of this study indicated that 231 (42.1%) of respondents had four and above ANC visits for recent delivery. Two hundred forty-three (44.3%) of the participants’ first ANC booking was at a place of their current childbirth health facility. About two hundred thirty-four (42.6%) of mothers’ recent childbirth were attended by a health care provider who had provided ANC services. Three hundred eighty-four (68.1%) of mothers were multi gravid. Of the total respondents, 408 (72.3%) of them gave birth with spontaneous vaginal delivery. Around287 (50.9%) mothers were delivered in the daytime, and 535(94.9%) were live birth (Table 2).

**Table 2:** Obstetric factors of mothers who gave birth in Hawassa city health facilities, southern, Ethiopia 2021 (*n* = 564)

Variable	Category	Frequency	Percentage
ANC follow up	Yes	549	97.3
	No	15	2.7
Place of first ANC booking	At the current place of childbirth	243	44.3
	Not at a place of childbirth	306	55.7
Number of ANC visit	One	77	14.0
	Two	81	14.8
	Three	160	29.1
	Four and above	231	42.1
Did your current birth attendant give you ANC	Yes	234	42.6
	No	315	57.4
How did you come to this health facility	Self-referral	349	61.9
	Institutional referral	215	38.1
Where did you plan to give birth	At the current place of childbirth	276	48.9
	Not at a place of childbirth	288	51.1
Place of current childbirth	public health hospital	273	48.4
	public health center	120	21.3
	private Institutions	171	30.3
Parity	Primipara	190	33.7
	Multipara	317	56.2
	Grandmultipara	57	10.1
Onset of labor	Spontaneous	445	78.9
	Induced	58	10.3
	Prelabor c/s	61	10.8
Labour stimulation	Yes	162	28.7
	No	402	71.3
Mode of last childbirth	Vaginal	408	72.3
	Instrumental	51	9.0
	Cesarean delivery	105	18.6
Length of stay at HF	less than 24 h	308	54.6
	24 or more hours	256	45.4
Newborn outcome	Alive	535	94.9
	Dead	29	5.1

### Person-Centered Care during childbirth scale and sub-scales

The respondents’ mean Person-Centered Care during childbirth score was 56 with a 95% Confidence Interval of (55.1, 57) and with SD ± 11.2 from 90. The minimum and maximum score for PCMCs was 30 and 82, respectively (out of 90).

**Fig. 1:**
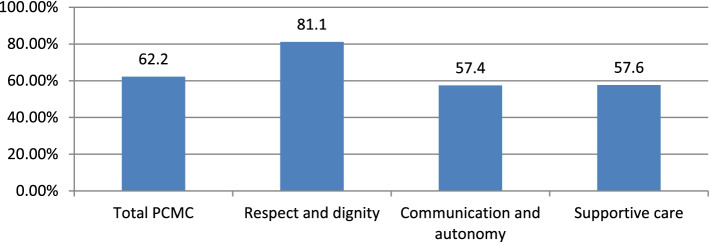
Percentage means a score of person-centered care full scale and subscales from the total expected score among mothers who gave birth in Hawassa city health facilities, Ethiopia, 2021

The formula made standardization of the mean score of actual score divided by potential maximum score times by one hundred. Therefore, the respondents’ percentages mean score of the total person-centered care during childbirth was 62.2% of the total expected score (Fig. 1).

### Distribution of full person-centered care during childbirth scale and sub-scales

Table 3 shows women had medium scores on the full person-centered care during childbirth scale. As shown in Table 4 Nearly a quarter of women had high person-centered care during childbirth score. Around 20.6% of respondents perceived respect and dignity as high.

**Table 3:** Descriptive statistics of person-centered care during childbirth among mothers who gave birth in Hawassa city health facilities, southern, Ethiopia 2021 (*n* = 564)

PCMC domain	Minimum	Maximum	Mean	Std. Deviation	Percentage		
					25^th^	50^th^	75^th^
Total PCMC score	30	82	56	11.2	48	56	64
Dignity and respect	3	18	14.6	5.7	13	15	16
Communication and Autonomy	7	20	15.5	2.39	12	15	19
Supportive care	11	41	25.9	6.6	21	26	31

**Table 4:** Total and subscale scores of person-centered care during childbirth among mothers who gave birth in Hawassa city health facilities, southern Ethiopia 2021 (*n* = 564)

Outcome variable	Frequency	Percentage
Full PCMC scale *N* = 564		
Low	131	23.2
Medium	299	53.0
High	134	23.8
Dignity and respect *N* = 564		
Low	93	16.5
Medium	355	62.9
High	116	20.6
Communication and Autonomy *N* = 564		
Low	105	18.6
Medium	341	60.5
High	118	20.9
Supportive care *N* = 564		
Low	122	21.6
Medium	318	56.4
High	124	22.0

### Factors associated with person-centered care during childbirth

In simple linear regression analysis, the residence of mothers, level of education, place of first ANC booking, number of ANC follow-ups, place of childbirth, childbirth which was attended by a care provider who had provided ANC service for mothers initially, plan for the place of childbirth, presence of labor stimulation and newborn outcome were significantly associated with person-centered care during childbirth. In multivariable linear regression analysis, the mother’s level of education, place of childbirth, childbirth which was attended by a care provider who had provided ANC service for mothers initially, and the newborn outcome was significantly associated with person-centered care during childbirth score (Table 5).

**Table 5:** Multivariable linear regression analysis factors for Person-Centered Care during childbirth, Hawassa city, southern Ethiopia, 2021(*n* = 564)

Variable	Category	Unstandardized Adjusted β Coefficients	95% CI of β
Residence	Urban	0	
	Rural	-1.01	(-2.84, .8)
Mothers education	No formal education	-3.00	(-5.27, -.73)*
	Primary school	-.59	(-4.28, -.94)
	Secondary school	-.48	(-2.55, 1.56)
	College and above	0	
Place of childbirth	Public health facilities	0	
	Private institutions	4.3	(2.37, 6.22)*
First ANC booking	A place of childbirth	1.03	(-1.24, 3.31)
	Not a place of childbirth	0	
Number of AVC visits	1–3	0	
	4 and above	.709	(-.95. 2.37)
Childbirth attendant	Attended by a care provider who had provided ANC for the mothers	5.48	(3.15, 7.81)*
	Attended by others who didn’t provide ANC for the mothers	0	
Plan for a place of delivery	A place of childbirth	0	
	Not a place of childbirth	.302	(-1.49, 2.09)
Labor stimulation	Yes	-.009	(-1.7, 1.68)
	No	0	
Newborn outcome	Alive	0	
	Dead	-7.0	(-10.4, -3.66)*

NB: * Statistically significant at *p* value < 0.05

## Discussion

This study attempted to establish the level of person-centered care during childbirth among women who gave birth in Hawassa city health facilities, Ethiopia. This study found that the mean score of person-centered care during childbirth was 56 with SD ± 11.2. The least score was on the communication and autonomy sub-scale, while the highest was on the respect and dignity dimension. This result is consistent with studies done in India, and Nigeria which showed that the mean score of person-centered care during childbirth was 55.8, and 55.13 respectively [17, 18]. The current finding showed that person-centered care during childbirth was low compared with the recommended scale value to say high person-centered care, which is above 75 percentile by the measurement tool validates and compared to other studies done in low- and middle-income countries [2, 12]. For this study the reason for low scale of person- centered care during childbirth finding might be attributed by Covid19 pandemic effect since the data for this study were collected during the pandemic effect of Covid19, which definitely affect the relationship between a care provider and mothers during the time of childbirth as well as the quality of the services [19, 20].

This finding indicates that mothers who didn’t get a formal education had decreased the person-centered care during childbirth (β = -3.00, 95% CI: (-5.27, -0.73). This result is in agreement with studies done in India and Kenya showing that college-educated women have a higher PCMC score than non-educated women [12]. A study done in peri-urban Kenya reported that literate women increase person-centered care levels during childbirth (β = 5.76, *p* = 0.006) [21]. The reason may be due to educated women may have better communication skills and can easily understand the situation in health institutions and may perceive better person-centered care than uneducated mothers. In addition, women’s education is also associated with improved health-seeking behavior through health awareness, economic autonomy, and the ability to make appropriate health decisions.

Mothers who gave birth at private health institutions increased the person-centered care during childbirth (β = 4.3, 95% CI: (2.37, 6.22). The result is in line with studies done in three resources limiting countries [12]. The current result is also in line with a study done in Dessie city showed that mothers who gave birth at a private health institution increased their person-centered care during childbirth as compared to their counterparts (β = 14, 95% CI: 7.70, 20.60) [15].

The reason for this finding might be due to private health institutions giving more emphasis on person-centered care to attract more clients and for sustainable utilization of their services. In addition, the quality of services providers of private health institutions may be better than that of public health institution providers.

Childbirth, which was attended by care providers who had provided ANC service for mothers, initially increased person-centered care during childbirth score (β = 5.48, 95% CI: 3.15, 7.81). This result is supported by the World Health Organization’s 2016 Midwife-led continuity of care models, which recommend a continuum of care with person-centered health care for pregnant women to improve positive pregnancy outcomes [22].

The reason for this might be because women who had ANC and discussed a place of childbirth were more likely to be familiar with the health care providers and has good communication and can easily understand each other if mothers gave birth at the same facility and especially if childbirth attended by a care provider who gave for Mother ANC service.

Mothers whose newborns dead was decreased the person-centered care during childbirth score (β = -7.04, 95% CI: -10.4, -3.66). The result is consistent with a study done in Dessie city indicating that respondents whose delivery outcome was dead had significantly lower person-centered maternity care [15]. The reason might be that mothers who lost their newborn may not be satisfied with the care given by the health professionals or might think that they lost their newborn due to poor care they received within the health institutions.

### Limitation of the study

Social desirability bias may be there because the participant may fear disclosing their negative experience during childbirth because of thinking of the service they gate may be affected if they come again to that health institution.

## Conclusion and recommendation

This paper showed that person-centered care during childbirth was low compared with other studies done in low- and middle-income countries. College and above level of mother’s education, giving birth at private health institutions and childbirth which was attended by a care provider who had provided ANC for mothers initially will improve person-centered care during childbirth. The Hawassa city health office should implement midwife-led care to improve person-centered care during childbirth, for researchers to conduct further research with large-scale studies is recommended, which will be important for policymakers to develop strategies and guidelines to apply the person-centered care during childbirth approach in maternity care units.

## Data Availability

The datasets used and/or analyzed during the current study are not publicly available because we did not have consent from all participants to publish raw data but are available from the corresponding author on reasonable request.

## Abbreviations

ANC: Antenatal Care
CI: Confidence Interval
HUCSH: Hawassa University Comprehensive and Specialized Hospital,
IDV: In Dependent Variable
PCC: Person-Centered Care
PCMC: Person-Centered Maternity Care
SD: Standard Deviation
SPSS: Statistical Package for Social Science
SSA: Sub-Saharan Africa
WHO: World Health Organization
